# The Impact of Psychological Factors on Women Entrepreneurial Inclination: Mediating Role of Self-Leadership

**DOI:** 10.3389/fpsyg.2021.796272

**Published:** 2021-12-23

**Authors:** Zhang Linfang, Rimsha Khalid, Mohsin Raza, Noppadol Chanrawang, Rehana Parveen

**Affiliations:** ^1^School of Business Administration, Southwestern University of Finance and Economics, Sichuan, China; ^2^Department of Business Management, Limkokwing University of Creative Technology, Cyberjaya, Malaysia; ^3^Faculty of Management Sciences, Phuket Rajabhat University, Phuket, Thailand; ^4^College of Law, Prince Sultan University, Riyadh, Saudi Arabia

**Keywords:** psychology factors, women entrepreneur, entrepreneurial intention, personality, self-leadership

## Abstract

The worth of women’s entrepreneurship is accepted globally, but there is less focus on it in developing countries, and societal expectations mean women often lack the confidence to start their own business. The core purpose of this research is to investigate the influence of personality traits on women’s inclination toward entrepreneurship. The personality traits are measured through the dimensions of openness, neuroticism, extraversion, conscientiousness, and agreeableness. Further, the study introduced the mediator of self-leadership on personality traits and entrepreneurial intentions of women. The study is quantitative in nature and used a questionnaire survey to collect the data by convenience sampling technique. The data was collected in the context of Pakistan, and Smart PLS was chosen for further analysis. The findings revealed the significance of the relationship between personality traits and entrepreneurial intention. Furthermore, the study also highlighted the significance of self-leadership as a mediator and proposed significant relationships. The study suggested that personality issues should be considered and used from a business perspective, and self-leadership is important for women. The study provides room for policymakers and institutes to inform educational policies to motivate women entrepreneurs for the future.

## Introduction

Currently, environmental change and urbanization patterns have prompted a developing discussion about balancing the standard of life through economic production in the future. Furthermore, entrepreneurship has become an essential issue; however, little research has explored entrepreneurship in this context (Bazkiaei et al., 2020). Additionally, numerous scientists (Antoncic and Hisrich, 2001) have noticed that a few factors like age, gender, and work insight—as individual factors—or family, companions, and education—as natural factors—have been found to impact entrepreneurial intentions. Research has also acknowledged the gender gap in business ventures and the significance of enterprising action for the economy (Boz and Ergeneli, 2014; Abdullah et al., 2018).

Specifically, in women, individual factors such as personality-related elements significantly affect women’s entrepreneurial intention. A few significant investigations have shown that psychological and character-related components are determinants of entrepreneurship’s aims (Tan et al., 2020). A complete understanding of the impacts of significant psychological elements on the attitude of women toward entrepreneurial aim would benefit national entrepreneurial education; however, few researchers have analyzed this topic (Antoncic et al., 2018; Bazkiaei et al., 2020; Palmer et al., 2021).

Some researchers have found that entrepreneurs carry some exceptional inborn elements, with those studies detecting a high level of heritability regarding entrepreneurial practices (Rauch and Frese, 2007; Nicolaou et al., 2008; Nicolaou and Shane, 2009; Duong et al., 2020; Elnadi and Gheith, 2021). Moreover, prior research suggests that business can be improved by analyzing the impact of personality characteristics in this regard.

In developing countries like Pakistan, the profile of women’s entrepreneurship has become an intriguing issue (Yousaf et al., 2021). Investigating the role of women in business, plus the obstructions and difficulties they confront, and the way they challenge the norms of being business visionaries, has been a key focus of the literature (Hussain et al., 2021; Khurshid et al., 2021). One of the main issues is a personality conflict that impacts women entrepreneurs in business. As with men, women entrepreneurs have different personalities; some may be suited to entrepreneurship, while others are not (Anwar et al., 2020). The problem may not arise due to good or bad personalities; instead, it may arise due to the conflict between different attitudes, irritability, cynicism, and arrogance, all of which contribute to negativity. A negative attitude may cause a disturbance in communication, creating a communication gap that may result in undesirable situations (Palmer et al., 2021). For example, a negative-minded person always sees all decisions negatively, and for this reason, others may not like to work with that person (Wang et al., 2016).

Similarly, issues may also arise due to competitive versus cooperative differences. This mentality can intentionally damage others’ professional lives, resulting in a loss of communication between the workers and hindering the business (Hueso et al., 2020). In this conflict, sometimes the stress becomes unbearable, causing workers to leave their jobs. The effectiveness of work entirely depends on teamwork. When this kind of negative mentality disrupts the team’s rhythm, the whole team suffers as does the work’s progression. Therefore, a person with a negative attitude can affect the morale of a group. Given the fundamental significance of business, the interplay between women entrepreneurs’ personality signifies a significant gap in the research (Boz and Ergeneli, 2014). The present study attempts to fill this gap.

This research, firstly, aims to examine the impact of the five factors model of personality traits on women’s entrepreneurial intention. Second, this research investigates the impact of the five factors model of personality traits on self-leadership. Third, this study examines the impact of self-leadership on women’s entrepreneurial intention. Lastly, this study investigates the mediating role of self-leadership between the five factors model of personality traits and women’s entrepreneurial intention. The study contributes theoretical knowledge regarding women’s entrepreneurship in developed and developing countries, especially in Pakistan. Theoretical research assists in this examination, through character attributes that comprise the five components of the five-factor personality model (extraversion, openness, neuroticism, conscientiousness, and agreeableness). Further, the self-leadership theory contributes by examining the self-leadership, which is described as an individual’s apparent capacity to perform tasks and accomplish objectives that bring about ideal results. Enterprising intention refers to one’s self-recognized conviction and foundation for setting up a new business, increasing the value of a current association, or deliberately intending to do so in the future. This study follows the reaming structure.

The introduction section explains the objective and significance of study; the second part is literature review that explains the theoretical and hypotheses development with previous studies. The third part is the methodology that contains information on the population, sample, and data collection procedures. Moreover, the fourth section is the analysis, results, and discussion. The final section, the conclusion, includes the theoretical and practical implications, and limitations of the study, along with suggestions for future research.

## Literature Review

### Women Entrepreneurial Intention

Entrepreneurial intention is necessary for financial development and is a significant component in lifting countries from poverty (Tomy and Pardede, 2020). Entrepreneurs are the main driver for financial development, work creation, and decreasing destitution in non-industrial countries. Entrepreneurship is a method to quicken economic development, and fast industrialization has been accomplished through this approach (Baluku et al., 2020; Nakara et al., 2020). Moreover, small businesses have been perceived as feeders administrating large scope ventures (Oladipo and Fabayo, 2012; Hu et al., 2018).

Siwadi and Mhangami (2011) revealed that women entrepreneurs are significant performers and supporters of financial development. They are becoming progressively more noticeable in the neighborhood economies of business-generating areas. Improving females’ financial and political strength has become a more prominent consideration in the last 30 years (Akinola Adeoye and Tella, 2013). Women’s entrepreneurship contributes widely through insights and the investment of a lot of energy and capital assets to their networks, plus they produce jobs and create extra work for providers and other side project business linkages (Polas et al., 2021). Women-owned organizations have expanded in recent years (Sajjad et al., 2020), and a developing measure of exploration shows that developing countries have neglected to address gender obstructions to critical economic development (Athanne, 2011). Nonetheless, the study discovered that men have a higher inclination for business behavior than females (Salahuddin et al., 2021). Women entrepreneurs have not had equal opportunities regarding their access to conditions and resources that encourage entrepreneurship, and at present, these boundaries confronting women in business have not been viably addressed (Athanne, 2011).

Therefore, Pakistan has a worthwhile opportunity in this context, as solving gender inequalities could create significant monetary development for the nation and women might want to attempt to start a business venture (Khalil et al., 2021; Noor et al., 2021).

### Personality Traits

The Five-Factor Model (FFM) of personality is a generally accepted personality model (Hofstee et al., 1992; Ariani, 2013). Zhao and Seibert (2006) demonstrated that the model’s development permits researchers to sort out different personality attributes into a useful schema to distinguish reliable connections. The FFM structure includes the five components of extraversion, openness, neuroticism, conscientiousness, and agreeableness. Research has confirmed that identity measures are substantial indicators of differences in employment criteria (Goldberg, 1993; Weisberg et al., 2011). Numerous dissimilar cognitive capacity measures and personality measures usually do not negatively impede workers (Hogan et al., 1996) and actually can upgrade decision-making processes. A recent examination demonstrated that personality measurements are related to work execution (Rosse et al., 1998; Dingemanse et al., 2010). The five identity measurements are: (1) neuroticism, a normal personality dimension indicating the general tendency to experience negative effects such as fear, sadness, embarrassment, anger, guilt, and disgust; (2) extraversion, including traits such as sociability, assertiveness, activity, and talkativeness, whereby extraverts are energetic and optimistic (Barrick et al., 1993); (3) openness to experience, which includes active imagination, aesthetic sensitivity, and attentiveness to inner feelings, a preference for variety, intellectual curiosity, and independence of judgment (Barrick et al., 1993); (4) agreeableness, whereby an agreeable person is fundamentally altruistic, sympathetic to others, and eager to help them, and in return, believes that others will be equally helpful (Barrick et al., 1993); and (5) conscientiousness, which refers to self-control and the active planning, organizing, and carrying out tasks (Barrick et al., 1993).

### Personality Traits and Women’s Entrepreneurial Intention

A person’s entrepreneurial intention could be affected by situational factors, such as the job’s characteristics, the organization, and co-workers (Strümpfer et al., 1998; Sahinidis et al., 2020) plus dispositional factors. Dispositional variables can be depicted as identity attributes, needs, demeanor, inclination, and thought processes that result in a propensity to respond to circumstances in a foreordained (inclined) way. Entrepreneurial intention is impacted by suitability, the requirement for accomplishment, regard toward oneself, locus of control, emotional demeanor, and association (Wu et al., 2019; Tan et al., 2021). Modern analysts have questioned the usefulness of personality measures in anticipating occupation-related criteria, in light of doubtful study results (Guion and Gottier, 1965; Ahmed et al., 2020) and are concerned that most personality measures are replicated. Personality traits are a multi-dimensional construct that demonstrate how well workers perform their undertakings, the actions they select, and the skill they demonstrate in tackling issues (Luc, 2020; Shah et al., 2020).

Moreover, it demonstrates the degree to which they finish actions, how they use their accessible assets, and the time and vitality they spend on their tasks (Neneh, 2020). Extraversion is described as a characteristic in which an individual is sociable, expressive, talkative, self-confident, bold, active, energetic, and determined. Entrepreneurs have a strong belief in having the ability to control their environmental outcomes. Extraversion has a positive effect on women’s entrepreneurial intention (Matlay, 2008). Moreover, past studies have demonstrated that openness is identified with effectively adjusting to change (Yap et al., 2012). Entrepreneurship involves one’s ability to investigate new ideas, utilize their imagination to tackle new issues, and apply creative ways to devise items, services, and business procedures. This characteristic also has a positive effect on women’s entrepreneurial intention (Zhao and Seibert, 2006). Conscientiousness comprises the particular characteristics of capability, request, loyalty, accomplishment endeavoring, self-restraint, and thought. According to these combinations, this characteristic also positively affects women’s entrepreneurial intention (Costa and McCrae, 1992).

Further, neuroticism comprises the particular qualities of tension, irate aggression, sorrow, reluctance, lack of caution, and weakness, which negatively affect women’s entrepreneurial intention (Costa and McCrae, 1992). Entrepreneurs normally work with low access to legitimate protection and a low margin for error in finance because they have restricted assets, and they are inclined to be serious, self-focused, and show low degrees of agreeableness (Zhao and Seibert, 2006; Postigo et al., 2021). In Pakistan, the examination has demonstrated that business visionaries or individuals with entrepreneurial intention regularly score highly on extraversion, openness, and conscientiousness, while relatively lower on neuroticism and agreeableness (Liang et al., 2015; Li et al., 2020). In this study, personality traits are used to examine women’s entrepreneurial intention. Therefore, the following hypotheses were formulated:

H1: Extraversion significantly influences women’s entrepreneurial intention.

H2: Openness significantly influences women’s entrepreneurial intention.

H3: Conscientiousness significantly influences women’s entrepreneurial intention.

H4: Neuroticism significantly influences women’s entrepreneurial intention.

H5: Agreeableness significantly influences women’s entrepreneurial intention.

### Relationship Between Personality Traits and Self-Leadership

Self-leadership has a wide range of theoretical sources. It works inside the structure of social, cognitive, self-regulation, motivation, self-management, self-influence, and self-efficacy theories. It coordinates these theories into comprehensive behavior and psychological strategies (Neck and Houghton, 2006). Self-leadership improves individual adequacy through explicit behavior and intelligent systems (Andressen et al., 2012).

Manz (1986) indicated that self-leadership is a thorough self-impact point of view that concerns driving oneself toward performance to start a business. It involves directing oneself to accomplish work that should be done but is not generally pressing. Self-leadership is characterized as a set of methodologies that address what could be done (objectives and standards) and why it is to be done (Manz and Neck, 1991). Self-leadership procedures might be separated into three general classes: behavioral focused strategies, natural reward strategies, and constructive thought pattern strategies (Neck and Houghton, 2006; Sarfraz et al., 2021).

Although self-leadership is conceptualized as intellectual conduct by Manz (1986), a few scholars (Houghton et al., 2004) have questioned whether self-leadership is a unique and recognizable idea, as for certain personality qualities, self-leadership may be a simple repackaging of individual characteristics clarified by prior and moderately stable personality traits. For example, Markham and Markham (1995) contend that one of the major hindrances of the self-authority hypothesis is its uniqueness compared to more conventional perspectives on comparable psychological sets. Similarly, Guzzo (1998) has asked whether self-leadership is distinguishable from other existing psychological constructs; for example, the personality measurement of conscientiousness. Markham and Markham further propose that it is conceivable that different parts of self-leadership merely reorganize past personality attributes. While some studies have endeavored to thoughtfully separate the self-initiative measurements from related psychological ideas (Neck et al., 1998; Houghton et al., 2003; Ho and Nesbit, 2018; Wu et al., 2019), the uniqueness of self-leadership and its measurements remain a topic for future study. Along these lines, the motivation behind the current examination is to experimentally research the degree to which self-administration speaks to a remarkable and important grouping of behavior and intellectual methodologies comparative with the impact of personality (Castellano et al., 2021). All in all, this investigation will analyze the degree to which self-leadership components are separate from personality factors and the idea of the connections between extraversion, openness, neuroticism, conscientiousness, and agreeableness variables. Therefore, the following hypotheses were formulated:

H6: Extraversion significantly influences self-leadership.

H7: Openness significantly influences self-leadership.

H8: Conscientiousness significantly influences self-leadership.

H9: Neuroticism significantly influences self-leadership.

H10: Agreeableness significantly influences self-leadership.

### Relationship Between Self-Leadership and Women’s Entrepreneurial Intention

The role of traditional leadership is still undergoing some changes globally (Rasdi et al., 2020). Leadership not only affects followers but also influences individuals in the firm. It helps to increase the individuals’ contribution to the organization (Khalid et al., 2020). Modern companies expect greater innovativeness, development, fast and adaptable activities, cooperation, and quick adaptability in an individual’s behavior. They also anticipate that their workers should show and enhance their authoritative capacities. In this structure, the executives or managers influenced by self-leadership also influenced the workers to develop their self-management skills for the organizations’ decision-making. Therefore, self-leadership ends up being very significant (Houghton et al., 2014). It improves individual capability through explicit social and psychological procedures (Andressen et al., 2012). Self-leadership techniques might be partitioned into three general classes: behavior-focused strategies, natural reward strategies, and constructive thought pattern strategies (Neck and Houghton, 2006).

The behavior-focused strategies incorporate self-perception, self-objective setting, self-reward, self-curing, and self-punishment. Self-perception helps collect accurate information about an individual’s practices, observations, or feelings and subsequent self-improvement. Additionally, it distinguishes practices that should be expanded or diminished and develops mindfulness about the reasons for those practices. In this way, people can efficiently oversee or assess themselves and eliminate or transform negative practices (Manz and Sims, 1980). Natural reward strategies depend on practices that underline the positive parts of a task to be finished. Natural reward strategies occur gradually and intrinsically, particularly when individuals manage various issues. Individuals attempt to handle the issues by driving circumstances instead of disregarding those issues while utilizing these strategies (Amundsen and Martinsen, 2015). Constructive thought pattern strategies include improving innovative thoughts or patterns to thoughts and making a propensity out of them that would impact an individual’s action (Anderson and Prussia, 1997; DiLiello and Houghton, 2006).

H11: Self-leadership significantly influences women’s entrepreneurial intention.

### The Mediating Role of Self-Leadership Between Personality Traits and Women’s Entrepreneurial Intention

This inner force is established in self-leadership skills (Pratoom and Savatsomboon, 2012); self-leadership addresses a blend of practices, perspectives, and thoughts that can drive oneself across testing and challenging conditions (Prussia et al., 1998; Houghton and Neck, 2002). Self-pioneers are bound to see themselves as skilled to perform at a more elevated level (Houghton et al., 2012), just as they apply themselves to accomplish the self-inspiration and self-direction expected to complete tasks desirably (Manz, 1992). Norris (2008) indicated that in conditions where representatives see support for driving themselves, self-leadership aptitudes might help amplify individual and expert qualities and execution.

Pratoom and Savatsomboon (2012) also recommended that when a society encourages risk-taking, the advancement of values, and supports learning by doing, self-leadership and the individual’s natural inspiration are emphatically influenced, therefore encouraging individual development. In this regard, the perception of a workplace that helps group creativity, risk-taking, and proactiveness, may encourage representatives who have self-leadership abilities to show inventive behavior.

When entrepreneurs are in the workplace, self-leadership aptitudes may add to the transformation of entrepreneurial intention into a day-by-day measure, either to make opportunities out of the ideal creative practices in the work environment or to eliminate components that block an individual’s entrepreneurial intention. In this way, self-leadership abilities give self-adequacy, inward motivation, self-impact, and mindfulness (Neck and Manz, 1996; Houghton and Jinkerson, 2007).

It might consequently be assumed that the perception of personality traits impacts the utilization of general self-leadership abilities, which influences women’s entrepreneurial intention. Investigating the mediating function of self-leadership adds to our knowledge regarding the idea of connections among personality traits, self-leadership, and women’s entrepreneurial intention. Appropriately, the accompanying hypotheses are constructed:

H12: Extraversion and women’s entrepreneurial intention are significantly mediated by self-leadership.

H13: Openness and women’s entrepreneurial intention are significantly mediated by self-leadership.

H14: Conscientiousness and women’s entrepreneurial intention are significantly mediated by self-leadership.

H15: Neuroticism and women’s entrepreneurial intention are significantly mediated by self-leadership.

H16: Agreeableness and women’s entrepreneurial intention are significantly mediated by self-leadership.

There is a developing collection of literature about self-leadership and its significance in the work environment, but few studies have satisfactorily analyzed the interceding function of self-leadership. According to Marques-Quinteiro and Curral’s (2012) research, mediation investigation supports the theory that self-leadership abilities bolster the connection between learning objective orientation and work role innovation, and partially intervene in the connection between characteristic inspiration and work role innovation. Another study found that self-leadership does not mediate between culture and collective individuals’ innovation (Pratoom and Savatsomboon, 2012). Also, to the best of the author’s knowledge, there has been no literature exploring self-leadership as a full or partial mediator of the connection between personality traits and women’s entrepreneurial intention. Baron and Kenny (1986) demonstrated that partial mediation is the most constant model in mindset research. Consequently, the partial mediation model is reasonable if the hypothesis and examination are vague about the intervention type.

Though, as per James et al. (2004), if hypothesis and exploration are lacking to theorize full or partial intervention, testing for full intervention is suggested since the full intervention model is the most appropriate mediation model. James et al. (2004) also demonstrates that full mediation should be an important or standard model in assessing mediation. In the current examination, personality traits have also been proposed to impact women’s entrepreneurial behavior through self-leadership skills. Consequently, the proposed theory was tried with a full mediation and contrasted with a fractional mediation model that incorporated the leading constructs’ potential direct impact.

### Theoretical Provision

#### Big Five Personality Traits Theory

Initially, the big five personality traits theory was developed and established by Fiske (1949). Later, this theory was expanded by some other researchers (Smith, 1967; Goldberg, 1981; McCrae and Costa, 1987). The five factors in the theory are extraversion, agreeableness, openness, conscientiousness, and neuroticism.

The general concept in normative social sciences is that women and men are more similar than different, but there are some exceptions. Therefore, Weisberg et al. (2011) studied the big five traits specifically in terms of personality and gender differences. The study found that women tend to score higher on extraversion, agreeableness, and neuroticism as compared to men. According to Weisberg et al. (2011) some personality traits are extensively separate in both women and men. This theory contributes in this current study to understanding the big five personality traits in women with entrepreneurial intention.

#### Self-Leadership Theory

Self-leadership is the practice of behavioral understanding, who you are, finding your preferred experiences, and intentionally directing yourself toward desired goals. The term was first developed from organizational management studies by Manz and Gioia (1983). Later, it was defined as “*comprehensive self-influence perception that directing oneself toward desired achievement by the naturally motivating tasks, also, management of oneself to do work that must be done but it is not motivating naturally*” (Manz, 1986). Moreover, this concept is based on the perception that self-leadership is a precondition for effective team leadership (Sims and Manz, 1991). In fact, more self-directing, self-leading individuals are more productive in their work role (Bendell et al., 2019).

**FIGURE 1:**
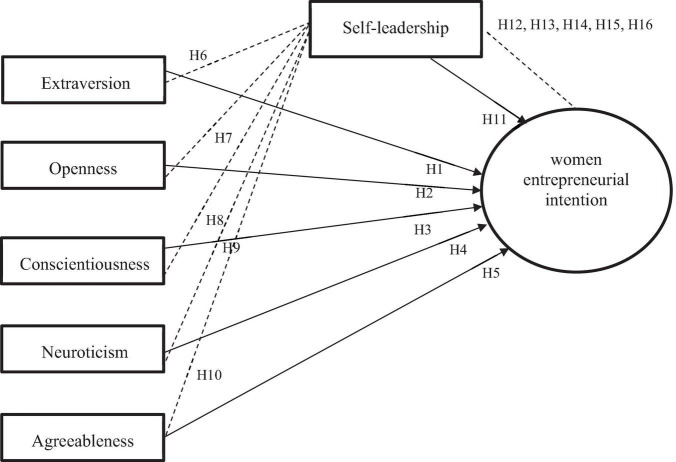
Conceptual model.

In this study, a conceptual framework based on the big five personality traits theory and self-leadership combines to explain women’s entrepreneurial intention. The big five personality traits theory is used to understand the big five personality traits as they relate to women with entrepreneurial intentions. Meanwhile, the self-leadership theory supports the notion that women use self-leadership practices to motivate themselves toward their desired goals. However, Figure 1 shows the graphical representation of the constructs.

## Methodology

### Data Collection

In this study, a survey form was used to investigate personality traits and women’s entrepreneurial intention. The survey form analyzed women entrepreneurs’ different personalities that affect business activities.

The quantitative approach was used in this study (Zechmeister et al., 1997). The target population of this study is female students in Pakistan. This institution also includes different education departments, so the researcher can get varieties of answers and different opinions on the nature of business. Data was collected from 350 female students, Kinnaird College for Women University, Lahore College for the Women University of Lahore, in Pakistan. Questionnaires were distributed to respondents but only 280 were received, of which 30 questionnaires were not adequately filled. The response rate is 65%. After that, 250 questionnaires were used in the analysis. The convenience sampling method was used to collect the data from female students. Data were collected electronically. The survey includes two categories of questions. One is demographic, and the other is personality traits, self-leadership, and women’s entrepreneurial intention.

### Measurement Scale

The quantitative data was designed in a questionnaire format. The respondents must answer all questions to assist progress in this quantitative data—an adapted questionnaire used as a research instrument to collect data for the research. The measurement of personality traits questionnaire was adapted by Farrukh et al. (2019). The measurement of women entrepreneurial intention constructs was adapted by Liñán and Chen (2009). In this study, the self-leadership questionnaire was adapted by Crossen (2015). Moreover, 5 Likert scales were used to measure the questionnaire. According to these questions, the researcher analyzed the different attitudes of women toward business activities.

## Results and Discussion

**FIGURE 2:**
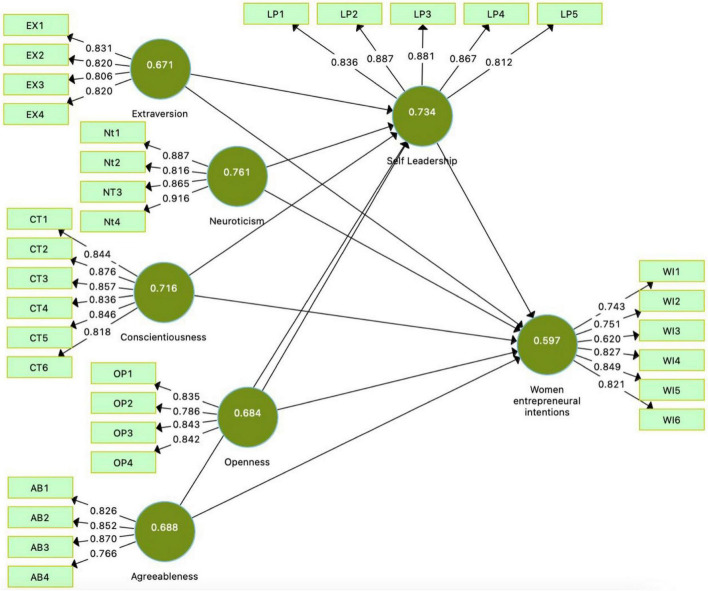
Measurement model.

**FIGURE 3:**
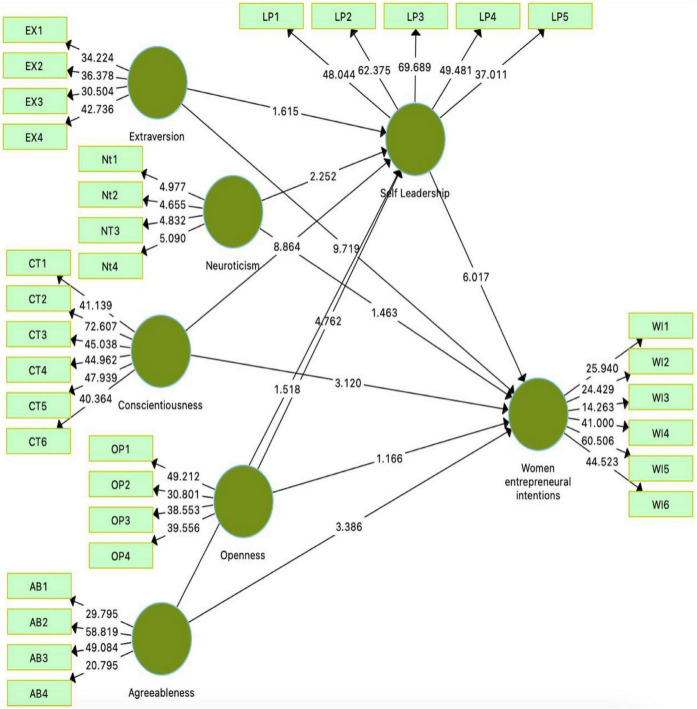
Structural equation model.

This study used partial least square and structural equation modeling for data analysis in Smart PLS (Hair et al., 2016). Smart PLS is an advanced tool for data analysis in measuring and assessing the model (Figures 2, 3). A total of 250 questionnaires were used in this study and considered satisfactory for the measurement and SEM (Reinartz et al., 2009).

### Demographics

Furthermore, 22.2% of students were 18–25 years of age, 26.5% of students were between the age of 26–35 years, 24.5% of students were in the age of 36–45 years, 26.8% of students were in the age of 46–55 years. Moreover, 25.9% of students had high school education, 21.9% of students had diploma education, 27.1% of students had a bachelor’s degree, 25.2% of students had higher education degree.

Table 1 shows the construct reliability and validity. The first step in reflex measurement is the internal consistency of the latent variable. We used alpha (α), compound reliability (CR), and Cronbach’s rho-A proposed by Hair et al. (2016) for internal consistency assessment. The thresholds for α, CR, and rho should be >0.70. The value of α, rho-A, and CR is greater than 0.70 and is considered satisfactory. In addition, this table also indicates the validity of convergence (AVE). The breakpoint is >0.50 value. The AVE values ranged from 0.597 to 0.761, which was considered good. In conclusion, this study did not find any internal consistency and convergence validity issues in the framework.

**TABLE 1:** Construct reliability and validity.

	Cronbach’s alpha	rho_A	Composite reliability	Average variance extracted (AVE)
Agreeableness	0.850	0.871	0.898	0.688
Conscientiousness	0.921	0.923	0.938	0.716
Extraversion	0.837	0.837	0.891	0.671
Leadership	0.909	0.911	0.932	0.734
Neuroticism	0.846	0.850	0.896	0.684
Openness	0.902	0.997	0.927	0.761
Women entrepreneurial intention	0.863	0.878	0.898	0.597
				

Tables 2, 3 show the discriminative validity of potential structures. Two methods can be used to assess discriminant validity: the Fornell and Larcker criteria proposed by Fornell and Larcker (1981) that is based on external load value, and the proposed Heterotrait-monotrait ratio (HTMT) ratio by Henseler et al. (2015). In contrast, HTMT is based on correlation values. The Fornell and Larcker criteria are considered an old method of evaluating discriminatory effectiveness, with a certain degree of sensitivity. The Fornell and Larcker method is the square root of the AVE value. All diagonal values of Fornell and Larcker methods should be greater than the rest of the relevant values (horizontal and vertical).

**TABLE 2:** Fornell–Larcker criterion.

	*A* *g* *r* *e* *e* *a* *b* *l* *e* *n* *e* *s* *s*	Conscientiousness	Extraversion	Leadership	Neuroticism	Openness	Women intention
Agreeableness	0.829						
Conscientiousness	0.433	0.846					
Extraversion	0.366	0.453	0.819				
Self-leadership	0.399	0.768	0.431	0.857			
Neuroticism	0.354	0.779	0.397	0.708	0.827		
Openness	0.039	0.068	0.066	–0.036	0.035	0.872	
Women entrepreneurial intention	0.481	0.655	0.655	0.678	0.538	–0.024	0.772
							

**TABLE 3:** Heterotrait–monotrait ratio (HTMT).

	Agreeableness	Conscientiousness	Extraversion	Leadership	Neuroticism	Openness
Agreeableness						
Conscientiousness	0.479					
Extraversion	0.429	0.513				
Leadership	0.433	0.838	0.493			
Neuroticism	0.401	0.880	0.470	0.807		
Openness	0.047	0.087	0.089	0.056	0.078	
Women entrepreneurial intention	0.567	0.712	0.776	0.737	0.607	0.041
						

On the other hand, HTMT has two thresholds based on free and conservative methods. This study uses a simple method to evaluate the discriminatory effects of the HTMT method. According to this method, the HTMT value should be <0.85 (Henseler et al., 2015). This study did not show discriminatory effectiveness.

### Quality Criteria

Table 4 indicates the quality criteria, which include the *R*^2^, Adjusted *R*^2^, *Q*^2^, and *F*^2^. This study measures the coefficient of determination by *R*^2^ value. Therefore, it shows the variance of dependent variables in the study because of predictor variables (Hair et al., 2014). The accepted criteria of *R*^2^ is 0–1, according to the value in this study that ensures the model’s predictive validity.

**TABLE 4:** Quality criteria.

	*R* ^2^	*R* ^2Adj^	*Q* ^2^	*F* ^2^
Self-leadership	0.638	0.633	0.461	
Women intentions	0.657	0.651	0.382	
Agreeableness → women entrepreneurial intentions				0.041
Conscientiousness → women entrepreneurial intentions				0.041
Extraversion → women entrepreneurial intentions				0.335
Neuroticism → women entrepreneurial intentions				0.109
Openness → women entrepreneurial intentions				0.106
Self-leadership → women entrepreneurial intentions				0.117
Agreeableness → self-leadership				0.108
Conscientiousness → self-leadership				0.249
Extraversion → self-leadership				0.113
Neuroticism → self-leadership				0.021
Openness → self-leadership				0.075

Further, the effect size is measured by (*f*^2^) that indicate the continuous relationship between constructs with multiple regression model (Cohen, 1988). The (*f*^2^) value range (≥0.02 is small; ≥0.15 is medium; ≥0.35 is large), according to the value of this study’s significant effect size between construct.

Other quality criteria of model (*Q*^2^) ensure the predictive relevance that indicates the predictive relevance in the structural model by the Stone–Geisser criterion. The accepted value of *Q*^2^ should not be more than 0 (Hair et al., 2013). According to the value of this study, the model confirms the predictive relevance.

Table 5 shows the direct and indirect effects of exogenous structures on endogenous structures. In this study, statistics show that while the results (beta = 0.394, *t* = 9.174, *P* = 0.000) indicate a significant impact between extraversion and women entrepreneurial intention. Therefore, H1 is accepted. Therefore, the H2 is rejected because the results (beta = 0.074, *t* = 1.129, *P* = 0.260) show an insignificant association between openness and women entrepreneurial intention. The study’s results found (beta = 0.224, *t* = 3.235, *P* = 0.001) that there is a significant impact of conscientiousness on women entrepreneurial intention. H3 is accepted. This finding is similar with (Zhao and Seibert, 2006).

**TABLE 5:** Direct and indirect effects.

Hypotheses	Beta	*T*-value	*P*-values
Agreeableness → self-leadership	0.061	1.468	0.143
Agreeableness → women entrepreneurial intentions	0.135	3.406	0.001
Conscientiousness → self-leadership	0.507	8.777	0.000
Conscientiousness → women entrepreneurial intentions	0.224	3.235	0.001
Extraversion → self-leadership	0.080	1.674	0.095
Extraversion → women entrepreneurial intentions	0.394	9.174	0.000
Neuroticism → self-leadership	–0.088	2.213	0.027
Neuroticism → women entrepreneurial intentions	–0.055	1.549	0.122
Openness → self-leadership	0.263	4.820	0.000
Openness → women entrepreneurial intentions	0.074	1.129	0.260
Self-leadership → women entrepreneurial intentions	0.332	5.887	0.000
Neuroticism → self-leadership → women entrepreneurial intentions	–0.029	2.054	0.040
Openness → self-leadership → women entrepreneurial intentions	0.088	3.518	0.000
Agreeableness → self-leadership → women entrepreneurial intentions	0.020	1.412	0.159
Extraversion → self-leadership → women entrepreneurial intentions	0.027	1.551	0.122
Conscientiousness → self-leadership → women entrepreneurial intentions	0.168	5.199	0.000

Neuroticism and women entrepreneurial intention have an insignificant relationship (beta = −0.055, *t* = 1.549, *P* = 0.122). H4 is rejected. Further, there is a significant relationship between agreeableness and women entrepreneurial intention (beta = 0.135, *t* = 3.406, *P* = 0.001) H5 is accepted.

Extraversion has an insignificant impact on self-leadership (beta = 0.080, *t* = 1.674, *P* = 0.095). H6 is rejected according to the results of this study. While, openness has a significant influence on self-leadership because results indicated (beta = 0.263, *t* = 4.820, *P* = 0.000), which shows significance as *t*-values are less than 1.96. H7 is, therefore, accepted. The study’s results (beta = 0.507, *t* = 8.777, *P* = 0.000) show a significant relationship between conscientiousness and self-leadership; H8 is accepted. The study findings found that neuroticism and self-leadership have a significant relationship (beta = −0.088, *t* = 2.213, *P* = 0.027). H9 is accepted. The results (beta = 0.061, *t* = 1.468, *P* = 0.143) indicate an insignificant impact of agreeableness on self-leadership. H10 is rejected. There is a significant impact of self-leadership on women entrepreneurial intention (beta = 0.332 *t* = 5.887, *P* = 0.000). H11 is accepted. The results of this study are consistent with (Houghton et al., 2004).

Self-leadership has an insignificant mediating relation between extraversion and women entrepreneurial intention (beta = 0.027, *t* = 1.551, *P* = 0.122) because some other factors are more influential with self-leadership. H12 is rejected. While, openness has a significant relation to women entrepreneurial intention with a mediating role of self-leadership (beta = 0.088, *t* = 3.518, *P* = 0.000); H13 is accepted. Conscientiousness has a significant relation to women entrepreneurial intention with a mediating role of self-leadership (beta = 0.168, *t* = 5.199, *P* = 0.000); H14 is accepted.

Therefore, neuroticism has a significant relation to women entrepreneurial intention with mediating effect of self-leadership (beta = −0.029, *t* = 2.054, *P* = 0.040); H15 is accepted. Furthermore, people with high neuroticism are more likely to experience depressive cognition leading to irrational belief patterns, reducing women entrepreneurial intention (Kirschenbaum, 1987; Pyszczynski and Greenberg, 1987; Cantor and Zirkel, 1990).

Self-leadership has an insignificant mediating relation between agreeableness and women entrepreneurial intention (beta = 0.020, *t* = 1.412, *P* = 0.159); H16 is rejected. These findings are consistent with previous research (Zhao and Seibert, 2006; Karimi et al., 2017).

## Conclusion

The study’s results conclude that extraversion, openness, and conscientiousness significantly influence women entrepreneurial intention. The findings also indicate that neuroticism and agreeableness are insignificantly associated with women entrepreneurial intention. Moreover, the study found that extraversion, openness, and conscientiousness significantly influence self-leadership while agreeableness has an insignificant relationship with self-leadership. In this study, neuroticism has a positive association with self-leadership. People with high neuroticism personalities are more likely to experience irrational belief patterns, resulting in reduced nervous consequences. Self-leadership is also significantly associated with women entrepreneurial intention. Further, the study shows that self-leadership significantly mediate the personality traits and women entrepreneurial intention.

### Study Practical Implications

This study gives the theoretical knowledge in literature in the context of women entrepreneurship. It contributes to the significance of the personality traits that affect women’s entrepreneurial intention. In the context of women entrepreneurs, women need to adopt a good workplace in the organization. The explanation behind this is to help the female working easily in business activities with new personalities. They should select only just the people who are strong on doing the work. There should be leaders with an open mind, and they ought not to be conservative. The enterprise could hold an intellectualizing meeting occasionally to know and comprehend each other very well. At times, a skilled worker could be a guide to an inexperienced one. In this situation, perhaps the organization can enlist more expert and experienced employees in their enterprises. The study will also assist in making the correct blend of old age and youth.

Further, self-leadership is considered a significant factor in entrepreneurial intention. The study helps women understand their personality traits according to business perspective and develop self-leadership skills according to their personality traits. Personality traits are natural factors, but self-leadership is not natural. It can be enhanced through actual practices. This factor will help women in enhancing confidence and assessing themselves from a business perspective. Self-leadership can help women in decision-making related to business activities. Furthermore, the study helps policymakers and institutions support women in developing these skills for innovative ideas.

### Recommendations

In this study, the five factors are extraversion, openness, neuroticism, conscientiousness, and agreeableness in women. Besides, these are a few suggestions for the organization with the goal that they can beat the issues. Government and policymakers should focus on individual personality issues and make strategies to help women deal with critical situations. They should offer some seminars to females for grooming their personalities from a business perspective. Educational institutions should teach subjects to females for personality issues and self-leadership in the perspective of business intention. They should adopt some practices for the development of entrepreneurial intention in females. Government and societies should create an open culture for females to think for themselves confidently and decide. The development of self-leadership in females enhance the decision-making form and help them to control their behavior in terms of environmental changes.

### Limitations and Future Research

The study focused on women entrepreneurs, and it should be conducted in future comparisons with men and women. It will be a better understanding of not just women but also men entrepreneurs. Furthermore, the study used self-leadership with personality traits in women entrepreneurial intention. It should be examined by other motivational factors with personality traits. This study is based on the big five personality traits theory and self-leadership theory, and it should be based on other motivational theories with personality traits. This study used a quantitative method to measure the factors. In future research, the advanced methodology should use these variables. Aside from this, the researchers also propose that different analysts do a similar examination on various working environments, for example, medical clinics, universities, and banks, on more members to draw nearer to accurate and precise information on the connection between their character and entrepreneurial intention.

## Data Availability Statement

The original contributions presented in the study are included in the article/supplementary material, further inquiries can be directed to the corresponding author.

## Ethics Statement

Ethical review and approval was not required for the study on human participants in accordance with the local legislation and institutional requirements. Written informed consent for participation was not required for this study in accordance with the national legislation and the institutional requirements.

## Author Contributions

All authors listed have made a substantial, direct, and intellectual contribution to the work, and approved it for publication.

## Conflict of Interest

The authors declare that the research was conducted in the absence of any commercial or financial relationships that could be construed as a potential conflict of interest.

## Publisher’s Note

All claims expressed in this article are solely those of the authors and do not necessarily represent those of their affiliated organizations, or those of the publisher, the editors and the reviewers. Any product that may be evaluated in this article, or claim that may be made by its manufacturer, is not guaranteed or endorsed by the publisher.
